# A qualitative description of birth trauma experiences from Ireland’s first psychological birth trauma clinic

**DOI:** 10.3389/fgwh.2025.1584070

**Published:** 2025-06-24

**Authors:** Anisha Bhagawan, Daria Prets, Ursula Nagle, Jillian Doyle, Richard M. Duffy

**Affiliations:** ^1^ Specialist Perinatal Mental Health Service, Rotunda Hospital, Dublin, Ireland; ^2^ General Adult Psychiatry, St Vincent's University Hospital, Dublin, Ireland; ^3^ UCD School of Medicine, University College Dublin Belfield, Dublin, Ireland

**Keywords:** birth trauma, perinatal, postnatal, triggers, PTSD

## Abstract

**Objective:**

Giving birth is a significant, life-transforming event that leaves lifelong memories. Although it is commonly seen as a beautiful and empowering experience, it can nevertheless also be traumatic and cause long-term psychological problems. Birth trauma, which arises from experiences during labour and birth, is gaining wider attention as a potential clinical issue. Our study aimed to categorize and describe qualitative data from women seeking assistance at a psychological birth trauma clinic.

**Methods:**

The study focused on analysing qualitative data from a psychological birth trauma clinic to understand women's perspectives and experiences. Thematic analysis was used for its flexibility and reliability. Out of 121 cases, 43 were excluded, resulting in 78 women included in the study.

**Results:**

This study identified three main themes: personal failure, failure of others, and threat to life; along with several sub-themes. These themes and sub-themes reflected the perspectives and experiences of the women in the study regarding their birth trauma experiences.

**Conclusion:**

This study emphasises the need for proactive measures to address childbirth trauma effectively, and advocates for Trauma Informed Care which promotes woman-centred practises to improve quality of care and health service delivery.

## 1 Background

The birth of a child is one of the most profound, life-changing experiences that can alter a woman's very sense of being and how she sees the world. Although most describe this experience as empowering and affirming, it can have a detrimental and traumatic effect on a woman's mental health (1–4).

Psychological birth trauma is increasingly recognized as a possible birth complication, defined by profound maternal psychological suffering and distress caused by intrapartum events (1, 5, 6). It is generally a subjective experience that takes place when a mother feels a threat to her own or her baby's physical well-being (4).

Psychological sequelae post-childbirth can be seen globally, across health care systems (7). There are many factors associated with post birthing distress including early experiences, the particular birth, how supported mothers were, and the surrounding environment (8). Such findings are consistent across multiple countries, including the UK, USA, Canada, and Australia, as well as from low- to middle-income countries such as Iran and Turkey. The literature reports that 20%–48.3% of women experience birth as being traumatic (9–11).

A traumatic childbirth is described as “a woman's experience of interactions and/or events directly related to childbirth that caused overwhelming distressing emotions and reactions, leading to short and/ or long-term negative impacts on a woman's health and wellbeing” (12).

A recent study conducted among mothers who delivered at an Irish Maternity Hospital found that 18% of mothers reported childbirth-related post-traumatic stress symptoms (CB-PTSS) after childbirth, despite only 4% of women meeting diagnostic criteria for childbirth (CB)-PTSD (9). Such findings support Grekin's research that not all traumatic childbirth journeys necessarily develop into clinical CB-PTSD (13).

As per the DSM-5, a diagnosis of PTSD occurs when symptoms from four distinct symptom clusters of criteria are present: re-experiencing cluster, avoidance cluster, negative cognitions and mood cluster, and arousal (14). There is clear evidence that CB-PTSS symptoms have adverse effects on women, even in the absence of threshold criteria for clinical PTSD (15). This could heighten concerns about future births (16), attachment to their newborn (17), and relationship difficulties with their partners (18).

### 1.1 The perinatal trauma clinic

Clinical services which provide psychological support to women who have experienced a traumatic birth in Ireland are very limited. An international mapping study which reviewed service-provision for traumatic birth across 18 European countries identified that only one third provided any type of psychological support, with varying degrees of quality (19). In 2020, a Perinatal Trauma Clinic was established within a large Irish maternity hospital, the first of its kind in the country. The clinic accepts referrals from GPs, community mental health teams, public health nurses, and various departments within the hospital. Any woman who has given birth at the maternity hospital can be referred if they exhibit trauma symptoms related to their perinatal journey encompassing antenatal, birth, postnatal, and neonatal care experiences. The PTC is led by an advanced midwife practitioner specialising in perinatal mental health and trauma, working collaboratively with a perinatal psychiatrist and clinical psychologist. The clinic accepts referrals for women any time in pregnancy and for up to one year postpartum.

All women who attend the service complete trauma screening measures. One of the measures – the self-assessment of maternal distress after a difficult birth (20), provides a narrative space for women to discuss their experience.

### 1.2 Aim

This study aimed to categorize and describe qualitative data from women seeking assistance at a psychological birth trauma clinic, in order to describe the context of birth trauma and the personal experiences of women.

### 1.3 Study design

This paper focused on the qualitative data collected from the PTC and attempted to use this information to examine the perspectives and experiences of women attending the clinic. Thematic analysis was employed to analyse the qualitative data as it is a flexible and robust methodology (21). Data was reviewed, analysed and coded. These codes were examined and themes identified (22).

A phenomenological methodology underpinned this analysis with the ‘object’ of human experience being the experience of a traumatic birth. This approach was adopted as we wished to primarily describe rather than explain the experiences of individuals. Additionally, we chose to take an inductive approach which identified patterns and themes within the data without any assumptions and proceeded to interpret these findings to extract valuable insights and comprehension (23, 24).

#### 1.3.1 Participants

**Figure 1:**
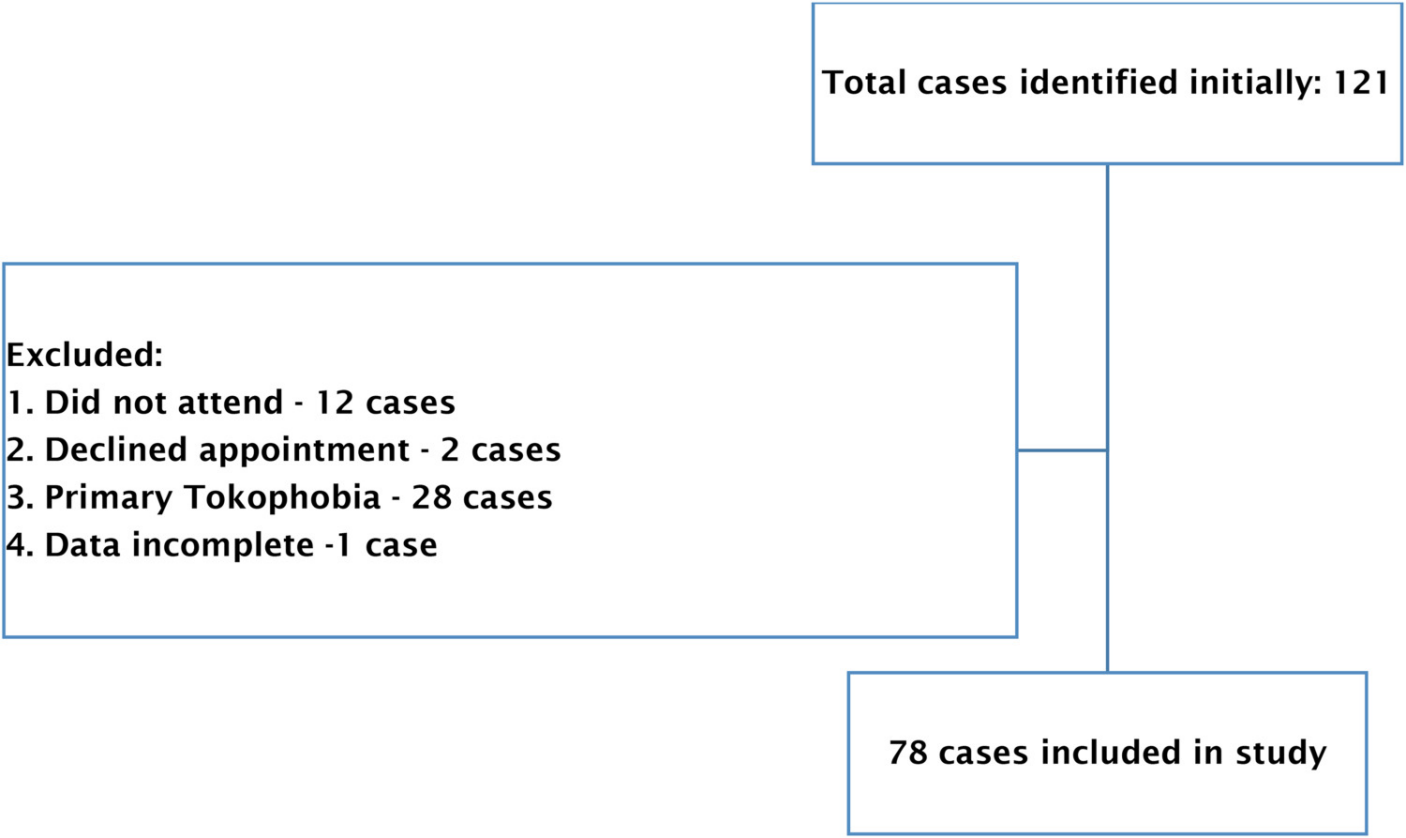
Individuals from the perinatal trauma clinic and their participation in this study.

All women who attended the PBTC between July 2020 and March 2022 were identified at initial stage of the study. Out of a total of 121 cases, 43 were excluded, resulting in 78 women being included in our study. The following cases were excluded: 12 cases due to non-attendance, 1 case where data was incomplete, 2 cases declined the appointment and 28 were primary tokophobia cases – these women attended our PBTC but were excluded as they did not have a prior experience of birth trauma to draw upon (Figure 1).

### 1.4 Data collection

Research data were collected retrospectively from the electronic patient records system. All data were anonymized. All women included in this study completed an initial assessment battery, which included a City Birth Trauma Scale (CBiTS) (25), Edinburgh Postnatal Depression Scale (EPDS) (26), Social Safeness and Pleasure Scale (27) and Self-Assessment of Maternal Distress after a Difficult Birth (20).

Demographic details and data relating to prior history, obstetric care, and neonatal factors were also taken from electronic patient records. The qualitative data that were analysed were extracted from the Self-Assessment of Maternal Distress after a Difficult Birth questionnaire from the answers to the four following prompts:

1. Positive memories from childbirth
2. Negative memories from childbirth
3. Concerns and fear for self
4. Concerns and fear for the baby's life

### 1.5 Data analysis

Qualitative data were transcribed in detail and organized in an Excel Worksheet. Two researchers, (AB and DP) independently processed the data by using MAXQDA, version 2022 software. To systemize and analyse qualitative data, the six-phase Thematic Analytical approach, proposed by Braun and Clarke was carried out (28, 29). These phases included familiarizing ourselves with the data, generating initial codes, searching for themes using Code Map and Visualisation tools, reviewing potential themes, defining and naming themes, and producing the report. A third researcher (RMD) reviewed the coding and resolved challenges with coding categorisation, this contributed to the iterative process of generating codes and identifying themes.

To enhance the credibility of the qualitative analysis the meeting of three researchers (AB, DP and RMD) was also used to reflect on personal biases and preconceptions in the research process. We also endeavoured to enhance the credibility through prolonged exposure to the area of birth trauma, four of the five authors worked with the trauma clinic outside of their research role for over a year and this experience helped them gain nuanced insights into women's experiences, behaviours, and beliefs (30).

Quantitative data was analysed using Excel, and frequency distributions, and means were calculated to provide descriptive data on study participants.

### 1.6 Ethical consideration

The studies involving human participants were reviewed and approved by Research Advisory Group, Research and Ethics Committee, Rotunda Hospital. The studies were conducted in accordance with the local legislation and institutional requirements. Written informed consent for participation was not required from the participants or the participants’ legal guardians/next of kin in accordance with the national legislation and institutional requirements.

## 2 Results

In this study, a total of 78 women participated, with an average age of 32.59 years. The demographic, obstetric, and neonatal characteristics of the individuals who attended the psychological birth trauma clinic are detailed in Table 1.

**Table 1:** Demographic, obstetric and neonatal characteristics of individuals attending the Perinatal Trauma Clinic (PTC).

	Characteristic	No/mean (SD)	%
Demographic variables	Total number	78	100
	Mean Age	32.59 (4.78)	
	Irish ethnicity	65	83%
	In relationship	70	90%
	Employed	71	91%
	Primipara	52	67%
	Planned pregnancy	65	83%
	Fertility treatment	10	13%
	History of mental illness	34	43%
	Prescribed medication for mental illness	14	18%
	Known to psychiatric services	15	19%
	In receipt of public health care	54	69%
Obstetric variables			
Type of labour onset	Spontaneous	21	27%
	Induced	43	55%
	Pre-labour section	14	18%
Mode of birth	Emergency low segment caesarean section	28	36%
	Planned low segment caesarean section	7	9%
	Spontaneous vaginal birth	18	23%
	Assisted birth	25	32%
Neonatal variables			
Gestational age at birth	Full term (37–40 weeks)	51	66%
	Premature (<37 weeks)	5	6%
	Postdates (>40 weeks)	22	28%
Neonatal ICU admission		23	29%

Table 2 shows the diagnostic criteria for PTSD in this cohort of women.

**Table 2:** Post traumatic stress disorder criteria Met on city birth trauma scale (DSM V).

Criteria	*N* (%)
A: Stressor	58 (74%)
B: Re-experiencing	72 (92%)
C: Avoidance	61 (78%)
D: Negative Cognitions & Mood	69 (88%)
E: Hyperarousal	55 (70%)
F: Duration	62 (79%)
G: Distress and Impairment	64 (82%)
H: Exclusion	2 (2%)
All Criteria	30 (38%)

### 2.1 Qualitative thematic analysis

Thematic analysis of the data generated three themes. Table 3 shows the main themes and subthemes that emerged. The three identified themes were feelings of personal failure, failure of others, and threat to life.

**Table 3:** Themes and sub themes identified from a thematic analysis of women attending the Perinatal Trauma Clinic (PTC).

Themes	Sub themes	Example quotes
Feelings of personal failure	Body failure	“I failed to deliver him on time… I could not progress”
	Emotional failure	“[I] felt so out of control and wasn’t aware of what was happening. I was not able to experience the birth properly. I feel I missed out on it”
	Failing the baby	“I knew there was something wrong. I couldn’t deliver him on time, and he ended up going to NICU”. “The detachment from baby at first”
Failure of others	Neglect and abandonment	“Feeling alone and unsupported during birth”
	Absence of dignity and privacy	“The doctor that removed my placenta didn't respect me” “feeling that the baby was ripped out of me” “lots of people around me”
	Dismissal of women's concerns	“Not being listened to by nursing staff”. “Being told by the midwife to go back to bed and I won't be getting checked because I'm not in labour…having to demand to get checked”. “Not receiving pain relief in labour”
	Lack of autonomy and decision making	“I felt forced into epidural which I didn't want. I was forced to give birth on my back”. “My baby being taken away without explanation”.
Threat to life	Maternal complications	“Seeing my husband crying and realizing something was very wrong…feeling I was dying”.
	Foetal complications	My baby's heart stopped in the labour ward”.

### 2.2 Theme one: feelings of personal failure

This theme consisted of three subthemes, as described below. While many women experienced feelings of failure within these subthemes, the differentiation was based on the focus of the perceived failure. One subtheme centred on individuals who believed they had failed their baby; while the other two subthemes revolved around women who felt that either their body or emotions had failed them.

#### 2.2.1 Body failure

In the study, sixteen women identified with the theme of experiencing a sense of failure or disappointment in their bodies following a challenging childbirth experience. Several participants expressed experiencing internalised feelings of failure when their expectations surrounding childbirth did not align with the reality of their birth experience. Some individuals grappled with emotions of inadequacy, failure, and guilt for not achieving the envisioned ideal birth, while others attributed their feelings of failure to the use of interventions or medications during labour.

For instance, one participant recounted a situation where she felt as though her body had let her down “being induced and on oxytocin caused my baby's heart rate to constantly drop. I wouldn’t have been induced if it were up to me. I felt like my body was failing me. [The clinician] had to use forceps to deliver her”.

Another woman blamed herself and expressed regret for not being able to have a natural birth and felt a significant amount of guilt as a result “I hoped for a natural birth but because I did not progress, I had to get a section even though I really didn't want to… [I have] a lot of guilt for it”.

#### 2.2.2 Emotional failure

Another factor contributing to perceived failure during childbirth was the emotional state of the women. Six women reported feeling disconnected from their bodies or experiencing a sense of unreality, which occasionally resulted in complete dissociation from the birthing experience. One woman expressed feeling “shocked and detached and can't remember the birth”.

Three women shared their challenges in forming an emotional connection with their baby, with some expressing feelings of failure in this regard. One woman, for example, described how she found it difficult to bond with her baby, stating, “I just couldn't bond with him… he felt like a stranger to me”. This woman also struggled with feelings of letting her baby down as she felt inadequate in fulfilling her role as a mother and could not establish that immediate loving bond with her baby “I felt very little love for him which made me question my abilities as a mother. I felt flawed as a mother’”. This highlighted the common overlap in these categories.

#### 2.2.3 Failing the baby

Fifteen women experienced feelings of failure towards their babies due to the decisions made during childbirth and the way their bodies responded.

One participant expressed regret over decisions made during childbirth, believing that different choices could have prevented distress for her baby “getting my water broken and the baby went into distress… maybe she would not have had to go through the distress if I made different decision”.

This sense of failure extended to the postpartum period for some women, who felt unable to care for their babies due to their own health issues or physical limitations. These feelings of failure had a significant impact on their self-worth and made them question their abilities as mothers. One woman shared “being sick myself, I could not care for my baby after birth. I could not do anything for her. She was choking on mucous, and I could not reach her, I couldn't soothe her on my own. I feel l have failed my child”.

### 2.3 Theme two: failure of others

This theme includes neglect and abandonment, dismissal of women's concerns, deprivation of dignity and privacy, and lack of autonomy and decision making.

#### 2.3.1 Neglect and abandonment

In this study, thirty-three women expressed their experience of a lack of emotional and practical support, which resulted in feelings of neglect and abandonment. They felt unheard in their communication of their needs, and emotions. For instance, one participant shared her frustration at not being fully informed about her condition and not receiving answers to her questions, which was distressing for her “[I] didn't fully understand what was happening. I was not informed about my condition”.

Another participant expressed feeling neglected and abandoned, stating she, “was left alone behind a curtain while in labour.”

Another woman described feeling intense loneliness and abandonment. “My fiancé was sent home, and I was left on my own. I could not see my family. I felt neglected by the medical staff”.

#### 2.3.2 Dismissal of women’s concerns

Twelve women felt that their needs, concerns, and opinions were dismissed by staff. They felt that their embodied experience of birth was disregarded or not taken seriously.

One participant shared that she felt dismissed and ignored for thinking she was in labour. Another woman felt that her midwife did not trust her to know her body and did not listen to her when she requested to be examined. She described “Not being believed that I was labouring and there was no one checking in. I progressed rapidly while getting from the toilet to the ward and ended delivering in the antenatal ward”.

Twenty-six women highlighted their focus on the experience of pain during childbirth. They reported instances where pain relief methods were not effective or when an epidural was not administered on time. These women expressed dissatisfaction with the pain relief. They identified the lack of sufficient analgesia as a significant factor contributing to their traumatic experiences.

One participant described being denied pain relief when requested it, “I was in such pain and agony… I repeatedly asked for an epidural but was told I was not far enough along”. Another woman stated that “the anaesthesia was wearing off. I could feel the pain during surgery and at them closing me up”.

#### 2.3.3 Absence of dignity and privacy

Eight women expressed concerns about the lack of privacy they experienced during labour. They were not informed about the identities of the professionals attending to them, which undermined their privacy. One woman specifically mentioned feeling a lack of privacy due to the presence of unfamiliar individuals entering and exiting the labour room “The number of people coming and going out of the room…there was no privacy”.

Another woman reported feeling violated due to multiple vaginal examinations conducted by healthcare professionals. This experience caused her to feel a sense of intrusion and discomfort “I felt violated..Felt like I was being poked and prodded”.

#### 2.3.4 Lack of autonomy and decision making

Twenty-five women shared their feelings of experiencing a loss of control over decision-making and autonomy during childbirth. They expressed a sense of their birth being solely controlled by the medical teams, leading to feelings of vulnerability and powerlessness.

For example, women stated, “I felt like my choice was taken away” and “I felt pressure to give birth naturally rather than c-section”.

### 2.4 Theme three: threat to life

#### 2.4.1 Maternal complications

Fifty women from our study reported experiencing a fear of dying and that those thoughts were usually in response to complications with the labour.

For example, one woman said that when she arrived at the hospital, she thought she “was dying because of uncontrolled bleeding”. Another woman said that she “developed heart failure and was unable to breathe”. She shared that this fear was so extreme that it led to fears of dying.

At times this fear was compounded by staff. One woman recalled “The frantic look of fear on the team. I was told I lost a lot of blood and may need a transfusion. I could feel my body slipping away. I thought I was dying.” This exacerbation of the fear was more direct with one woman reporting “I was told by a doctor that I could die giving birth and so could the baby”

#### 2.4.2 Foetal complications

The fear of death that the participants expressed was not only related to themselves but also to that of their babies. Fifty-two Women reported worries, such as, “When she was born, and they took her away as she was in distress; she was taken to NICU straight away. I knew there was something very wrong”.

Another participant also described the moments before her child was born and the fear that she felt at that point: “I developed pre-eclampsia and there was loss of foetal heart rate…had to go to theatre to save baby. [I] Didn't think we were going to live through this”.

## 3 Discussion

It is important to recognize that women from racial minority groups and low-income populations often experience higher rates of trauma during childbirth (31, 32). Notwithstanding that the majority of our participants were in relationships (90%), employed (91%), had planned pregnancies (83%), were adult women (mean age 32.59) and had not experienced the stressors associated with immigration and cultural isolation (83% white Irish) and thus could be seen to have a number of psycho-social and demographic factors considered protective against birthing trauma; a large number of these patients nonetheless reported DSM V symptoms of psychopathology and birthing trauma. It is thus reasonable for national service commissioners to assume that the rate of birthing trauma could even be far higher in catchment areas that service more marginalised and vulnerable women, where the relative presence of these psycho-social and demographic protective factors are lower.

Women with pre-existing severe mental illness typically are at a higher risk for adverse pregnancy and neonatal outcomes (33). Research by Boden et al. have found that both untreated and treated women diagnosed with bipolar disorder had elevated risks for caesarean sections, assisted deliveries, non-spontaneous onset of delivery, and premature births (34). Amongst our participants it was found that 43% of women had a history of mental illness. Our findings demonstrated that 45% of participants underwent a caesarean section, with 36% of these being emergency procedures, a rate significantly higher than that observed in the general population (35). Furthermore, 32% had assisted deliveries. Dekel et al. found that women who underwent obstetric interventions, such as caesarean sections or instrumental vaginal deliveries tend to experience greater psychological distress after childbirth compared to those who had natural or vaginal deliveries (36). There may thus be merit in service managers proactively identifying women with known vulnerabilities or risk factors such as a history of mental health problems or the need for obstetric interventions associated with birthing trauma, for access to enhanced care interventions.

The literature indicates that traumatic childbirth is more prevalent among women whose infants needed Neonatal ICU (NICU) care. Specifically, the stress associated with having a baby in the NICU has been associated with symptoms of postpartum PTSD (37).

In our sample, 29% of infants required NICU care, suggesting a significant portion of the participants may have experienced additional and under recognised stress and potential trauma related to their childbirth experiences requiring appropriate supports. Again, this is a subject that health care providers may need to be more mindful of and to accord more careful monitoring of.

Interestingly while a significant portion of the participants perceived childbirth as traumatic, only 38% met the clinical criteria for PTSD. Notwithstanding not making the threshold for a formal diagnosis of PTSD, these women reported high levels of distress and sub- clinical symptoms, which require compassionate interventions within a patient centred model of care.

The complex nature of childbirth experiences and the varying degrees of trauma that women may encounter during this vulnerable time can have lasting effects on daily life, relationships and future pregnancies (38, 39). This highlights the importance of recognizing and addressing the emotional impact of childbirth on women's mental health, regardless of meeting diagnostic criteria. Healthcare providers need to be sensitive to recognizing and responding empathetically to these experiences, ensuring that women receive the necessary support and care during this critical phase (12). By acknowledging and addressing these emotional challenges, healthcare providers can help promote the well-being and mental health of new mothers.

Each of the key-subthemes identified, together with related clinical practice recommendations, are examined in the context of the existing literature below.

### 3.1 Feelings of personal failure

These responses highlighted the significant and enduring impact that feelings of failure can have on women during the childbirth process. Research by Kjerulff and Brubaker revealed that women who experienced unplanned caesarean births are more likely to feel disappointed and view themselves as failures compared to those who had spontaneous vaginal births (40). Furthermore, they also found that women who had unplanned caesarean births had less positive overall feelings about their childbirth experience in comparison to women who had spontaneous vaginal or planned caesarean births. This indicates a link between unplanned caesarean births and negative emotional responses. These adverse emotions, encompassing fear, lack of control, anger, disappointment, guilt, and feelings of failure, can significantly impact the overall birth experience of women and may lead them to perceive the event as traumatic. Our study indicated a rate of 36 percent of women who had experienced an unplanned caesarean section perceived their birth as negative and traumatic, which supported this finding.

To anticipate and attempt to minimise feelings of failure, open discussions about women's birthing expectations and the development of informative interventions tailored to their specific needs, as recommended by Hollander et al. are essential (41). Creating a supportive environment where women can express their expectations and where healthcare providers can clarify the current clinical situation and communicate any deviations from the original plan in real-time, may empower women with the knowledge to avoid self-blame by better understanding the reasons behind the clinical decisions made. Clinicians must be mindful of their language, as terms like “failure to progress” can exacerbate feelings of failure in women.

A recent systematic review which examined the current evidence on trauma-informed care practices and policies in maternity services has highlighted the importance of clear, collaborative and sensitive communication between healthcare professionals and women and their families (42). Fostering this approach in maternity services empowers women to be actively involved in decision-making processes involving their care. Healthcare professionals can have an opportunistic role in reducing or preventing traumatic birth experiences through the provision of respectful, supportive care and communication which ensures women feel safe (7, 41, 43). Our research findings indicated that many participants expressed feelings of failure in their roles and actions, with some also sharing struggles in forming attachments and feelings of love towards their babies.

It is generally uncommon for women to be separated from their babies at birth; however, in this particular group, such separations were quite common, often occurring due to either health consequences to the mother, or problems with the baby. Mothers who have experienced this early separation may grieve the loss of those crucial first moments with their newborn, leading to feelings of guilt and a sense of inadequacy in fulfilling their baby's needs. These feelings can greatly affect how women perceive themselves as mothers and lead many to feel that they have let their child down. This aligned with previous research by Schneider (44).

### 3.2 Failure of others

During the birthing process, women place immense trust in healthcare professionals, relying on them to alleviate fears and support the well-being of both mother and baby. Consequently, substandard care during labour, lack of support, and ineffective communication from healthcare professionals can leave expectant mothers feeling powerless, isolated, and anxious. Women who receive support from both their loved ones and healthcare professionals feel more empowered, which enables them to express their thoughts and emotions openly, leading to a more positive birthing experience (45).

Healthcare professionals have historically made decisions based on their perception of “best interests”, without adequate consideration of an individual's rights, will and preference. This dynamic is shifting, however in the context of childbirth individuals still describe situations where healthcare providers make unilateral decisions without adequately involving them, or considering their emotions, wishes, or apprehensions (46).

In cases of traumatic birth, a paternalistic approach can result in feelings of powerlessness, fear, and emotional trauma. The absence of control or involvement in a significant and personal event can deeply affect a woman's mental and emotional well-being (47). Factors such as privacy, trauma-informed care, and the presence of past sexual trauma are essential considerations, as individuals with a history of trauma may be particularly vulnerable to experiencing birth trauma.

In 2014, the World Health Organization (WHO) recognized obstetric violence as a major and serious public health concern that impacts women's healthcare rights throughout pregnancy, childbirth, and the postpartum period (48). Since then, there has been a growing interest in understanding obstetric violence and its impact on women's mental health. This has led to an increase in research publications and collaboration among medical professionals from various disciplines, including psychology and social work, in an effort to address the issue of obstetric violence and its effects on women's mental health. Obstetric violence encompasses any mistreatment or disrespectful behaviour that a woman may encounter from healthcare providers. This can include instances of being ignored, neglected, yelled at, touched without consent, coerced into procedures during labour, or not being provided with sufficient information to make informed decisions. Obstetric violence can have serious repercussions on a woman's mental and physical well-being, potentially resulting in trauma, depression, or a reluctance to seek medical treatment in the future (49).

Reducing traumatic birth experiences often involves shifting towards a woman -centred approach. It is essential to engage in collaborative decision-making with the mother. Effective communication and advocacy are key components in this approach. By empowering women to actively participate in decision-making during childbirth, the likelihood of traumatic experiences may be significantly reduced, fostering a more positive and supportive birthing environment (12). There is thus an identified need for maternity services to move towards implementing a trauma-informed approach to maternity care to minimise or prevent traumatic birth experiences (7, 42, 50). The concept of trauma-informed care has emerged in healthcare as a systematic approach to reduce the risk of re-traumatisation (10). Introducing a trauma-informed approach to maternity services means that all staff within an organisation develops an understanding and recognition of the impact of trauma on service-users, families and all staff within their organisation (7, 51, 52). Further research is needed to identify best-practice approaches for implementing this paradigm (53, 54).

Labour pain is considered one the most intense forms of physical pain that women may encounter during their lifetime. Effective pain management plays a crucial role in the holistic care provided during childbirth when requested. While epidural analgesia is often considered the gold standard method for labour analgesia, various factors such as patient preferences, contraindications, limited availability, and technical challenges may prompt the consideration of alternative pain management strategies, including pharmacologic and non-pharmacologic modalities (55). In our study, inadequate pain management emerged as a significant factor contributing to traumatic experiences. Hodnett proposed that offering supportive care from caregivers could help alleviate pain, supported by a Cochrane review indicating that continuous support during labour can decrease the requirement for pain relief, and lead to more positive birth experiences (56).

### 3.3 Threat to life

The experiences reported by participants in our study align with previous research by Hollander indicating that fear and concerns about potential harm to oneself or one's baby can contribute to a traumatic birth experience (41). The impact of NICU admission on maternal anxiety, as highlighted by Baum, underscores the emotional toll such situations can have on mothers (57). Therefore, this suggests that the fear of harm to oneself or one's child during childbirth can significantly influence the experience of birth trauma, with distress being reported long after birth. These findings emphasized the importance of continuous psychological support for women during this vulnerable period. In our study, the focus on evaluating trauma symptoms may have prompted participants to recall life-threatening situations that may not have been as salient in their memory before the assessment.

### 3.4 Clinical implications

The findings of our study emphasized significant emotional, psychological, and systemic challenges that women face during childbirth. These experiences highlighted the need for a shift towards care practices that are trauma-informed, compassionate, and centred on women (58). Clinicians should be attentive not only to the physical aspects of childbirth but also address the emotional and psychological needs of women. Improved and transparent communication, shared decision-making, especially in high-risk situations, can help to alleviate feelings of helplessness and minimize trauma (59). In addition, care models that offer continuity, such as midwife-led care, the presence of a birth partner or family member, have been proven to enhance outcomes and satisfaction. In relation to respect for dignity, privacy, and bodily autonomy, staff should be trained to always introduce themselves, explain procedures, and ensure that examinations are conducted respectfully with consent. Considering that many women described ongoing psychological distress linked to their birth experiences, seamless referral pathways to perinatal mental health services must be established, particularly for women expressing guilt, fear, or emotional disconnection (60). In terms of health policies, our findings underscored the need of incorporating respectful care standards into national guidelines, take steps to eradicate obstetric violence, ensure access to perinatal mental health services, and support care models that prioritise continuity and patient autonomy. Furthermore, investing in staff training, clear communication protocols, routine evaluation of women's experiences and quality improvement programs are crucial to establishing a maternity care system that is both clinically effective and emotionally safe (58, 59).

### 3.5 Strengths

This study analysed data collected from Ireland's first perinatal trauma clinic. Such research has never been previously done in an Irish population. By incorporating both qualitative and quantitative data, the study offered a more comprehensive description of the participating cohort. To ensure the trustworthiness and credibility of the qualitative research, the study adapted the stages and criteria outlined by Walsh and Downe (61).

### 3.6 Limitations

The study did not have access to information regarding prior exposure to trauma. Its design focused solely on collecting variables at the point of intake into the clinic, which may limit its ability to reflect trauma symptoms more broadly over time, as trauma symptoms can emerge later. Additionally, the individuals attending the clinic are not representative of all women who have given birth, as the clinic was in a specific maternity hospital serving a specific geographical catchment area. Women with severe symptoms of avoidance may not have sought treatment at the clinic, despite having traumatic birth experiences. The analysis conducted in this study was based on free text answers, which, while limited in detail, allowed for the inclusion of a large cohort of women. This approach helps identify key topics for future exploration, although it may not have provided the depth of understanding that other forms of qualitative research could. Additionally, the content of some of the qualitative answers may have been influenced by the answering of other questionnaires during the same time frame.

## 4 Conclusion

Our study contributes to the growing body of evidence on women's traumatic birthing experiences in Ireland. The focus on feelings of personal failure, failure of others, and threat to life in childbirth trauma sheds light on important aspects of women's experiences. The global recognition of the effects of traumatic birthing as indentified by our study highlights the need for proactive measures to address this issue effectively. Transitioning to holistic and humanistic care approaches can play a significant role in improving the overall psychological well-being of women. Examples of such interventions include antenatal preparation, early assessment of mothers at risk of developing psychological birth trauma and ensuring appropriate training for maternity and medical staff to enhance their ability to provide emotional support and adequate care during the childbirth process. Perhaps more pressing, is the need for maternity services to move towards adopting a trauma-informed approach to care provision within maternity services. While this is not a simple undertaking, healthcare systems require a strategic and systemic approach to implementing practices that reduce the risk of re-traumatisation to services users, families and staff.

## Data Availability

The original contributions presented in the study are included in the article/Supplementary Material, further inquiries can be directed to the corresponding author.
